# Simulation of Graphene Field-Effect Transistor Biosensors for Bacterial Detection

**DOI:** 10.3390/s18061715

**Published:** 2018-05-25

**Authors:** Guangfu Wu, M. Meyyappan, King Wai Chiu Lai

**Affiliations:** 1 Department of Mechanical and Biomedical Engineering, City University of Hong Kong, 83 Tat Chee Avenue, Kowloon, Hong Kong, China; guangfuwu1987@gmail.com; 2 Centre for Robotics and Automation, City University of Hong Kong, 83 Tat Chee Avenue, Kowloon, Hong Kong, China; 3 Shanghai Key Laboratory of Modern Optical System, Engineering Research Center of Optical Instrument and System (Ministry of Education), University of Shanghai for Science and Technology, Shanghai 200093, China; 4 NASA Ames Research Center, Moffett Field, Mountain View, CA 94035, USA; m.meyyappan@nasa.gov

**Keywords:** simulation, graphene field-effect transistor, biosensors, *Escherichia coli*, motion

## Abstract

Foodborne illness is correlated with the existence of infectious pathogens such as bacteria in food and drinking water. Probe-modified graphene field effect transistors (G-FETs) have been shown to be suitable for *Escherichia coli* (*E. coli*) detection. Here, the G-FETs for bacterial detection are modeled and simulated with COMSOL Multiphysics to understand the operation of the biosensors. The motion of *E. coli* cells in electrolyte and the surface charge of graphene induced by *E. coli* are systematically investigated. The comparison between the simulation and experimental data proves the sensing probe size to be a key parameter affecting the surface charge of graphene induced by bacteria. Finally, the relationship among the change in source-drain current (∆*I_ds_*), graphene-bacteria distance and bacterial concentration is established. The shorter graphene-bacteria distance and higher bacterial concentration give rise to better sensing performance (larger ∆*I_ds_*) of the G-FETs biosensors. The simulation here could serve as a guideline for the design and optimization of G-FET biosensors for various applications.

## 1. Introduction

The extraordinary electrical properties of graphene, characterized by its high carrier mobility and conductivity, have driven exploration of applications in nanoelectronic devices [1,2], including biosensors. Graphene field-effect transistors (G-FET), in particular, have been extensively explored as a promising platform for bio-species detection [3]. The existence of infectious pathogens such as bacteria is correlated with human foodborne diseases. There are two major considerations for the design and operation of a G-FET based biosensor. First, an aqueous solution is necessary to provide the liquid condition for the operation of G-FET biosensors. Over the past several years, most of the reports on graphene field-effect transistors have addressed operation under vacuum or atmospheric conditions. Recently, the operation of graphene in aqueous electrolytes, for potential use in biosensors and bioelectronics, has been reported [4,5]. The use of solution-gated epitaxial graphene as a pH sensor was first demonstrated by Loh et al. [4]. Ohno et al. then reported electrolyte-gated graphene field-effect transistors for detecting pH and protein adsorption [5]. Second, it is necessary to functionalize the graphene surface to introduce the sensing probe needed for biosensing. The surface functionalization of graphene with aromatic molecules in different organic solvents has been studied by analyzing their influence on the carrier mobility of graphene, which helps to select proper solvents [6]. Moreover, functionalization of graphene with sensing probes equips the biosensors with the specific detection ability. In previous studies, graphene was functionalized with antibodies, aptamers, or nanoparticles acting as the sensing probe to develop biosensors with high selectivity and sensitivity [7,8]. Although excellent performance has been achieved experimentally, understanding the G-FET operation is key to further development and optimization for various applications.

There have been a few reports on modeling and simulation of FET-based biosensors. The Incremental Support Vector Regression (ISVR) algorithm was employed to study the aptamer-modified G-FETs for interferon-gamma (IFN-γ) detection, in which the change of Dirac voltage and the shift of transfer characteristics were mathematically modelled and simulated [9]. Recently, the simulation of G-FETs for DNA hybridization has attracted some attention [10,11]. A graphene-solution interface capacitance model was developed to analyze the change in graphene-solution quantum capacitance (*C_q_*) when DNA hybridization occurred on the graphene surface, with results yielding more than 97% accuracy [10]. Another model on the DNA adsorption on the graphene surface used particle swarm optimization (PSO), where the graphene surface was modified by single-stranded DNA (ssDNA) and then the source-drain current (*I_ds_*) was simulated when the graphene biosensor was exposed to the complementary DNA [11].

Previously, we have demonstrated G-FETs for *Escherichia coli* (*E. coli*) detection by using antibody [12] or aptamer [13] sensing probes, showing *E. coli* concentration-dependent electrical response. More importantly, the biosensors exhibited rapid detection of *E. coli*, with a response time of about 120 s. Here, the motion of *E. coli* cells in solution and the surface charge of graphene induced by *E. coli* are modeled and simulated with COMSOL Multiphysics to interpret the operation of graphene biosensors. The size of the sensing probe proves to be the important factor affecting the efficiency of the induced charges on the graphene surface; the smaller-sized sensing probe affords more efficient induced charges on the graphene surface. Additionally, the relationship between the bacterial concentration and the electrical current of the graphene biosensor is established in this work, showing that higher bacterial concentration gives rise to larger electrical response (larger source-drain current, ∆*I_ds_*), and the saturation is correlated with the active sensing size of graphene and the bacterial size. The simulation of the G-FET biosensors here could help to understand their operation and pave the way to designing and optimizing new biosensors with better sensing performance.

## 2. Model Description

### 2.1. Structure of the G-FET Biosensor

**Figure 1:**
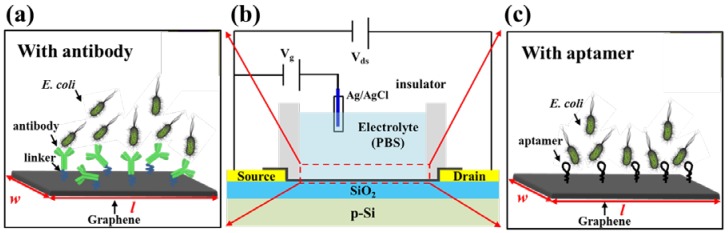
The general schematic of the probe-modified G-FET biosensor. (**a**) linker/antibody-functionalized G-FET for *E. coli* detection. (**b**) general structure of the G-FET biosensor. (**c**) aptamer-functionalized G-FET for *E. coli* detection.

The general schematic of the sensing probe-modified G-FET biosensor is shown in Figure 1. For a typical solution-gated G-FET (Figure 1 middle), there are three terminals: source, drain and gate electrodes. The source and drain electrodes were prepared on Si wafer with a 300 nm oxide layer. 15 nm Cr and 90 nm Au were deposited on top of the Si chip to form the contact pads, which were connected by patterned graphene. The source and drain electrodes were then insulated to prevent any possible leakage current. The active sensing area of graphene was about 100 µm (width) × 100 µm (length). The sensitive layer of the G-FETs consists of the linker and the sensing probe (the antibody as shown in Figure 1a or the aptamer as shown in Figure 1c). Based on the AFM measurement results, the heights of the whole linker/antibody and aptamer were about 5 nm and 3.6 nm, respectively [12,13]. PBS (phosphate buffer saline) buffer (0.1 mM, pH = 7.4) was used as the detection electrolyte [6]. A PDMS (polydimethylsiloxane) chamber was assembled with the G-FET to confine the electrolyte.

### 2.2. Bacterial Motion in Solution

**Figure 2:**
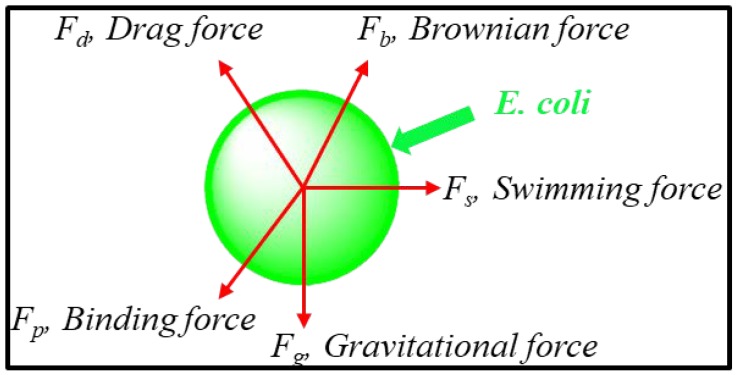
The motion of *E. coli* in the electrolyte affected by different forces: gravitational force (*F_g_*), Brownian force (*F_b_*), drag force from the liquid (*F_d_*), binding force created by the sensing probe (*F_p_*) and the force resulting from *E. coli* swimming in electrolyte (*F_s_*).

When the G-FET biosensor was exposed to different concentrations of *E. coli*, the detection time was about 2 min. We assume that when a droplet of *E. coli* solution is added to the PDMS chamber, *E. coli* cells move in the solution. When the cells are close to the graphene surface, they would be sensed by the biosensor. The process from the addition of *E. coli* solution to the stability of the detection signal could take 2 min. Generally, the motion of *E. coli* in the electrolyte is affected by different forces, which include gravitational force (*F_g_*), Brownian force (*F_b_*), drag force from the liquid (*F_d_*), the binding force created by the sensing probe (*F_p_*) and the swimming force created by *E. coli* (*F_s_*) as shown in Figure 2.

According to Newton’s second law [14], the force balance on a moving particle (consider *E. coli* cell as particle) is described by

(1) $$m_{p}\frac{d{\vec{u}}_{p}}{dt}={\vec{F}}_{g}+{\vec{F}}_{b}+{\vec{F}}_{d}+{\vec{F}}_{p}+{\vec{F}}_{s}$$

#### 2.2.1. The Gravitational Force

Considering buoyancy, the gravitational force can be expressed as:

(2) $$F_{g}=-V_{p}g\left(\rho_{p}-\rho \right)$$

where *V_p_*, *ρ_p_*, *ρ* and *g* are the volume of the particle, the density of the particle and fluid, and the acceleration due to gravity, respectively.

#### 2.2.2. Brownian Force

Random collisions of molecules of the fluid with the suspended particles cause a random movement called Brownian motion. The Brownian force can be estimated as [15]:

(3) $$F_{b}=\zeta \sqrt{\frac{12\pi \kappa_{B}\mu Tr_{p}}{\Delta t}}$$

where *κ_B_* is the Boltzmann constant, *µ* is the viscosity of the liquid, *T* is the absolute temperature, *r_p_* is the radius of the particle, and Δ*t* is the magnitude of the characteristic time step. The parameter *ζ* is a Gaussian random number with zero mean and unit variance.

#### 2.2.3. Drag Force

For a particle suspended in a fluid flowing under conditions of low Reynolds number, the drag force is estimated from Stokes’ law and the relative velocity [16]:

(4) $$F_{d}=3\mu \pi d_{c}\left(u_{s}-u_{p}\right)$$

where *µ* is the viscosity of the fluid, and *u_s_* and *u_p_* are the velocities of the fluid and the particle, respectively. The apparent diameter of the composite particle *d_c_* can be estimated based on the size of the *E. coli* cell.

#### 2.2.4. Binding Force

The interaction between a sensing probe such as an antibody (or aptamer) and *E. coli* is mainly based on electrostatic attraction. Considering the sensing probe and *E. coli* as two charged particles, the binding force between them can be expressed as

(5) $$F_{p}=k_{e}\frac{q_{1}q_{2}}{r^{2}}$$

where *k_e_*, *q*_1_, *q*_2_ and *r* are the Coulomb’s constant (8.99 × 10^9^ N·m^2^/C^2^), the signed magnitudes of the sensing probe and *E. coli*, and the distance between the sensing probe and *E. coli*.

#### 2.2.5. Swimming Force

*E. coli* can swim in the solution, which will generate a swim force for its motion. The swim force (*F_s_*) is given by

(6) $$F_{s}=\frac{d\left(m_{p}u_{p}\right)}{dt}$$

where *m_p_* is the mass of *E. coli*, and *u_p_* is the velocity of *E. coli*.

#### 2.2.6. Other Forces

In addition to the forces discussed above, other forces acting on a particle in a liquid also exist, such as particle-particle interaction forces, van der Waals attraction force, thermophoretic force, lift force and magnetic forces between particles [17]. Depending on the type of separation phenomena, these forces can be added to Equation (1) resulting in a complex model, which can only be solved by numerical simulation. When the bacteria cells move to the surface of graphene, some of them will be captured by the antibodies or the aptamers due to the high affinity between them.

### 2.3. Electrostatic Gating Mechanism

**Figure 3:**
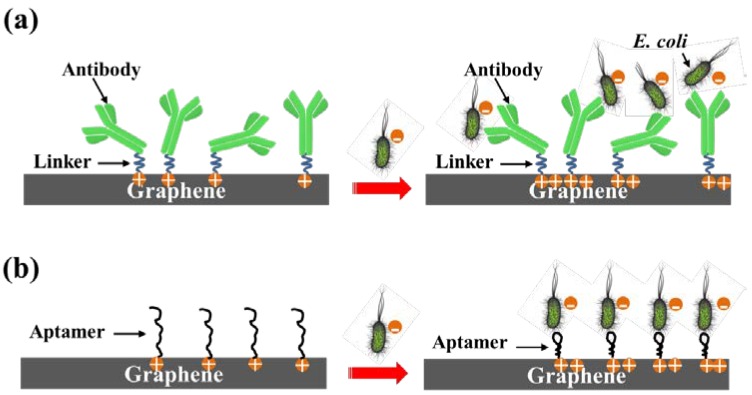
(**a**) Electrostatic gating with antibody as the sensing probe. (**b**) Electrostatic gating with aptamer as the sensing probe.

Since graphene is electrically grounded, the negatively charged *E. coli* imposes an external electric field with direction towards the electrolyte. The external electric field shifts the Fermi level of graphene downwards. Therefore, when the negatively charged *E. coli* is added and captured by the sensing probe (antibody or aptamer), the hole carrier density of the graphene increases, as shown in Figure 3. As a result, the right-shift of the Dirac point (p-doping) and the increase of *I_ds_* in the operating region (p-region) of the transfer characteristics are observed from the experimental results [12,13].

### 2.4. Gauss’s Law

Because of the long-range nature of Coulombic interactions [18], electrostatics plays a fundamental role in virtually all interactions involving biomolecules in ionic solution [19]. Continuum models of molecules in ionic solutions, first proposed in 1923 by Debye and Hückel [20], are important tools for studying the electrostatic interaction in ionic solutions. Gauss’s law relates the distribution of electric charge to the resulting electric field. In free space or vacuum, Gauss’s law may be expressed in its differential form

(7) $$\nabla \cdot E(r)=\frac{\rho (r)}{\varepsilon },$$

where ∇ denotes divergence, *E*(*r*) is the electric field, and *ρ*(*r*) is the total electric charge density on the surface, and *ε* is the free permittivity of space. Using the relation

(8) E(r)=−∇Φ

where Φ is the scalar potential, Equation (8) can be rewritten as:

(9) $$\nabla \cdot \nabla \Phi =\nabla^{2}\Phi =-\frac{\rho (r)}{\varepsilon }$$

The dielectric constant or relative permittivity, *ε_r_*, is defined as the ratio of the permittivity in the dielectric, *ε*, to the permittivity in vacuum, *ε*_0_:

(10) $$\varepsilon_{r}=\frac{\varepsilon }{\varepsilon_{0}}$$

Finally, the differential form of Gauss’s law for a dielectric can be written as

(11) $$-\nabla \cdot \left(\varepsilon_{r}\cdot \varepsilon_{0}\nabla \Phi \right)=\rho (r)$$

## 3. Simulation Methods

Equations (1) and (11) were solved here by the Finite Element Method (FEM)-based commercial software COMSOL Multiphysics to visualize the time-dependent bacterial motion in the electrolyte and the charge distribution on the surface of graphene induced by charged *E. coli* cells.

### 3.1. Simulations of Bacterial Motion Using COMSOL

#### 3.1.1. Setup

**Figure 4:**
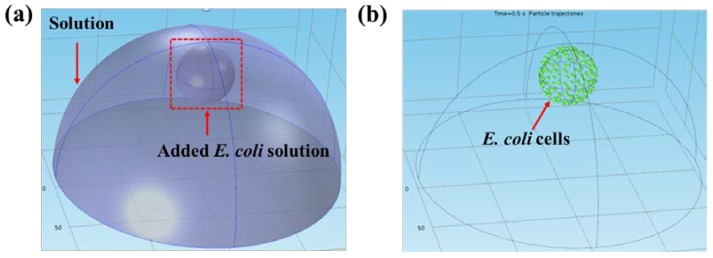
The setup of bacterial motion in solution. (**a**) The structure of the solution and the bacteria droplet. (**b**) The initial state of *E. coli* cells in the droplet.

The input parameters for the simulation were taken from the experiment in [13]. As shown in Figure 4a, the large half ball is the solution confined by the insulator on the top of graphene, and its radius is about 3.63 mm. The small ball located inside of the half ball is the added droplet of *E. coli* solution. The radius of the droplet is about 1 mm. The smaller green particles are *E. coli* cells as shown in Figure 4b. The radius of *E. coli* cells is approximately 0.5 µm. The duration of particle motion in solution is set at 120 s, which is the same as the real-time electrical current monitoring experiment. Adding *E. coli* solution at different concentrations allows the number of *E. coli* cells close to graphene surface to be simulated and counted.

#### 3.1.2. Bacterial Distribution

**Figure 5:**
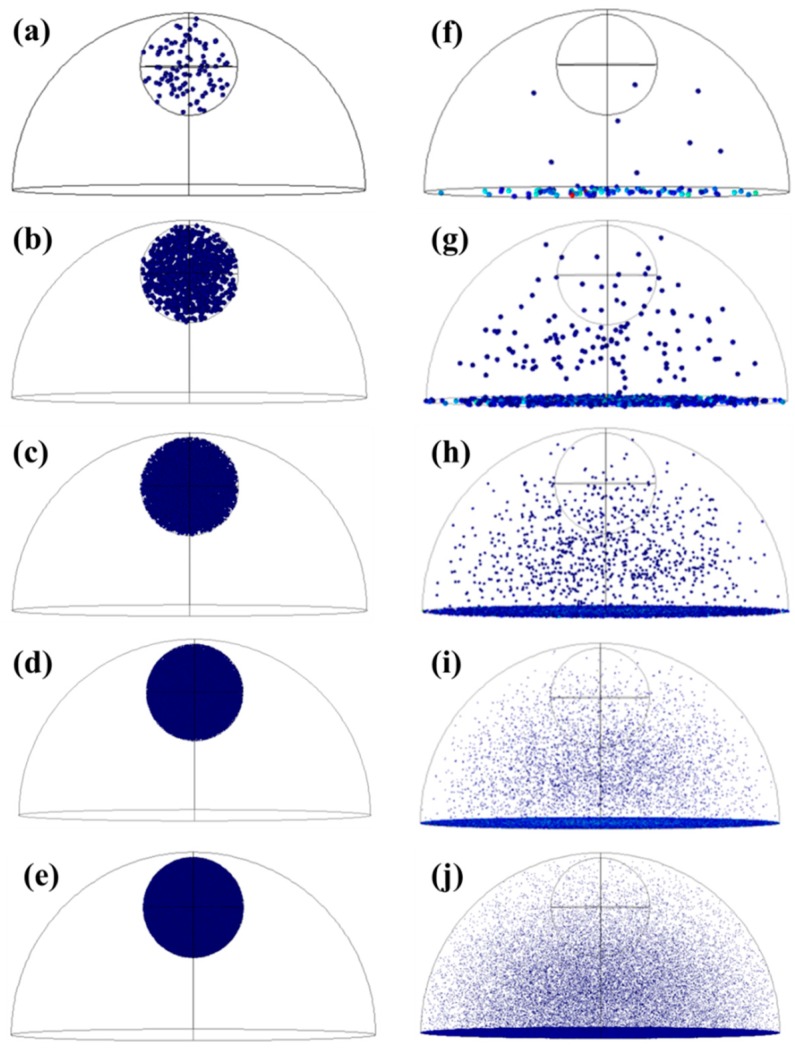
The distribution of different particle concentrations at *t* = 0 and *t* = 120 s. (**a**–**e**) The initial state of 10^2^ (**a**), 10^3^ (**b**), 10^4^ (**c**), 10^5^ (**d**), and 10^6^ (**e**) particles added in the electrolyte. (**f**–**j**) The distribution of 10^2^ (**f**), 10^3^ (**g**), 10^4^ (**h**), 10^5^ (**i**), and 10^6^ (**j**) particles in the electrolyte after 120 s.

**Figure 6:**
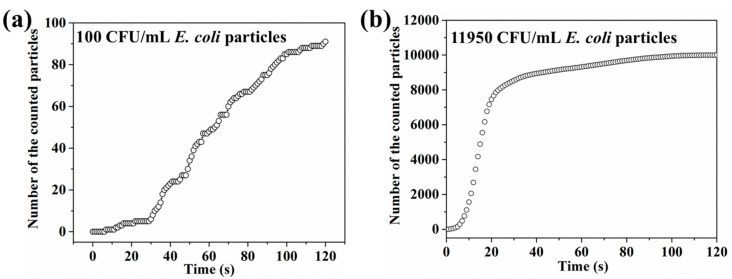
(**a**) The bacterial distribution from 0 to 120 s when 100 CFU/mL *E. coli* was simulated. (**b**) The bacterial distribution from 0 to 120 s when 11,950 CFU/mL *E. coli* was simulated.

Based on the real-time current monitoring for different bacterial concentrations [13], the particle concentration was set as 10^2^, 10^3^, 10^4^, 10^5^ and 10^6^. The distribution of the particles at different concentrations is shown in Figure 5 and the counted number of the particles on the bottom at different concentrations is shown in Table 1. Most of the particles reached the bottom for all five concentrations. Moreover, the number of particles on the bottom exhibited time-dependent motion. As shown in Figure 6a, when 100 particles were added, the number of particles on the graphene surface increased slightly in the first 30 s followed by a much more significant increase from 30 to 120 s. However, when 11,950 particles were simulated, as shown in Figure 6b, the particles reached the graphene surface within a shorter time. The slight increase of the bacterial number on the graphene surface was obtained in the first 10 s, followed by a significant increase from 11 to 90 s with a final saturation in the last 30 s. The larger percentages for 10^3^ and 10^5^ CFU/mL concentrations may be due to the interactions between bacterial particles. Similarly, when 10^3^ CFU/mL bacterial particles was simulated, the number of particles on the graphene surface increased slightly in the first 20 s followed by a much more significant increase from 20 to 120 s. However, when higher concentrations of bacterial particles (10^4^, 10^5^, and 10^6^) were simulated, most particles reached the graphene surface within a shorter time (~20 s), and a saturation behavior could be observed for all three higher concentrations. Detailed information can be found in the supporting information (Figure S1). The simulation of the bacterial particle motion in electrolyte will help us to understand the current change in the experiment.

### 3.2. Simulation of the Surface Charge in COMSOL

#### 3.2.1. Setup

**Figure 7:**
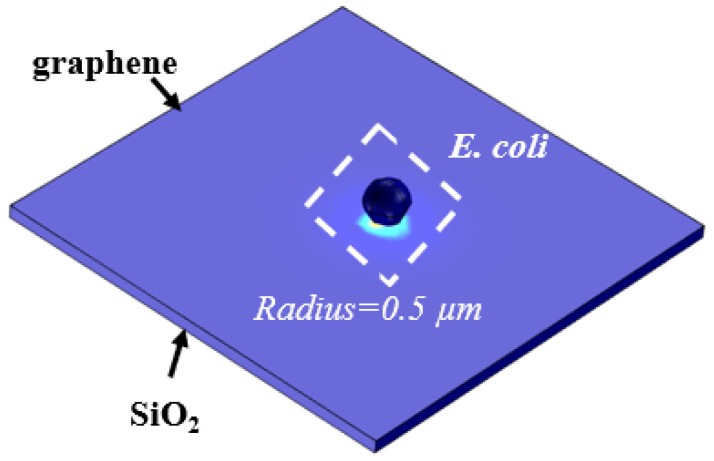
The setup of the charged particle on the graphene surface.

The induced charges by the charged particles on the surface of graphene can be simulated using the electrostatic simulation model in COMSOL. The dark sphere in Figure 7 is the *E. coli* particle with a radius of about 0.5 µm. Both the width and length of graphene are 10 µm. The graphene film is on top of a 300 nm SiO_2_ layer. The surface charge of *E. coli* in PBS buffer is about −3 × 10^−16^ C [21]. According to Equation (11), the charge on graphene surface induced by the charged *E. coli* particles is influenced by the dielectric material and the distance between graphene and *E. coli*; here, we mainly focus on the effect of the distance on the induced charge on the graphene surface.

#### 3.2.2. Surface Charge Induced by *E. coli* Particle

**Figure 8:**
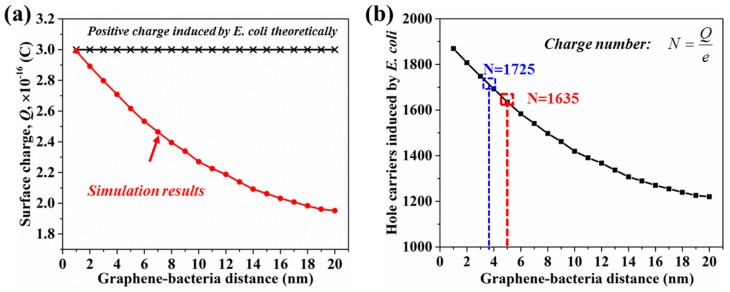
(**a**) The simulated surface charge induced by a single *E. coli* particle at different graphene-*E. coli* particle distances. (**b**) The relationship between the hole carriers induced by a single *E. coli* particle and graphene-*E. coli* distance.

We simulated the surface charge induced by negatively charged *E. coli* particle at different values of graphene-*E. coli* particle distance (*d*). Theoretically, when a particle containing −3 × 10^−16^ C charges is located on a plate, positive charges of 3 × 10^−16^ C will be induced on the surface of the plate. In our simulation, the induced charges exhibit a distance-dependent decrease as shown in Figure 8a. The surface charge induced by a charged *E. coli* particle decreases when the graphene-*E. coli* particle distance increases, which indicates that the efficiency of charge inducement will decrease when large-sized sensing probes are employed as the sensing component. The hole carriers induced by a single *E. coli* particle is calculated by

(12) $$N=\frac{Q}{e}$$

where *N* is the carrier number; *Q* is the surface charge; *e* is the elementary charge (1.6 × 10^−19^ C). As shown in Figure 8b, the number of hole carriers induced by single bacteria decreases when the graphene-*E. coli* particle distance increases. As the heights of the linker-antibody complex and pyrene-tagged DNA aptamer are 5 nm and 3.6 nm, the hole carriers induced by single *E. coli* are 1635 and 1725, respectively. We used these two values in the following calculation.

## 4. Change of Source-Drain Current vs. Bacterial Concentration

Based on the simulation results of bacterial motion and the hole carriers induced by single *E. coli* cell, we calculated the source-drain current change (∆*I_ds_*) when the graphene device was exposed to various concentrations of *E. coli*. As discussed above, the source-current modulation is expressed as a function of the change in the carrier density (∆*N*) in the graphene channel, which is proportional to the number of targets attached on the graphene surface [13]:

(13) $$\Delta I_{ds}=\frac{w}{l}\cdot e\cdot \mu \cdot V_{ds}\cdot \Delta N$$

where *w* is the width of the graphene channel; *l* is the length of the graphene channel; *e* is elementary charge (1.602 × 10^−19^ C); *µ* is the carrier mobility; *V_ds_* is the source-drain voltage.

The binding of the negatively charged *E. coli* induces more holes in the graphene channel. Based on Equation (13), the net change in source-drain current (∆*I_ds_*) caused by *E. coli* was evaluated at the given *V_g_* (0.04 V) and *V_ds_* (0.05 V). The carrier mobility (1030.99 cm^2^/V·s) for the source-drain current calculation was obtained from our previous experimental results [13]. Several factors could affect the carrier mobility of a graphene device including the substrate, temperature, charged impurities, defects on graphene film and graphene-metal contact [22]. Substrates that possess a similar lattice constant to graphene and an atomically flat surface are ideal for preserving the intrinsic carrier mobility of graphene. The mobility will be reduced by increasing the temperature due to the increased scattering that depends on the acoustic phonons. Charged impurities and vacancies on graphene will cause Coulomb scattering and short-range scattering, respectively. Both factors will reduce the carrier mobility of graphene. Non-ohmic graphene-metal contact will damage carrier injection, which will also reduce the carrier mobility of the graphene device. Considering these factors, the substrate, temperature and graphene-metal contact were kept stable during our experiments. Only the attachment of charged impurities (namely bacterial particles) might affect the carrier mobility of G-FET, here. However, the bacterial particles were captured by the sensing probes (antibody or aptamer) on the graphene surface, which could screen the scattering effect from bacterial particles. Moreover, since the total detection time is about 10 min in our experiments, the reduction of carrier mobility of graphene is negligible within such a short time. The comparison of transfer curves before and after exposure to high concentration of bacterial solution and the estimation of carrier mobility were further performed to prove this assumption. Analysis of the slopes of the transfer characteristics before and after addition of bacterial solution (10^6^ CFU/mL) reveals (Figure S2) negligible changes (Detailed information can be found in supporting information). Thus, a fixed carrier mobility was used in the calculations.

**Figure 9:**
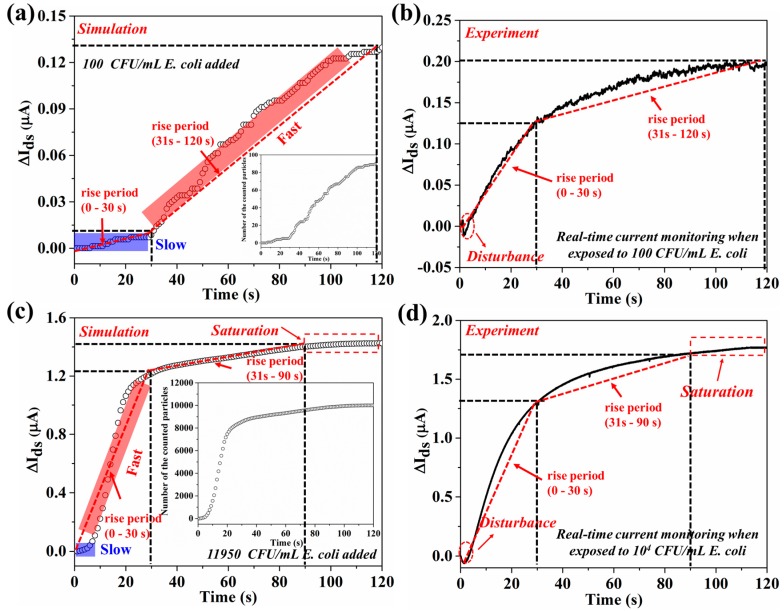
The relationship between ∆*I_ds_* and *E. coli* motion in the simulations and experiments. (**a**) The ∆*I_ds_* vs time when the simulation was performed with 100 *E. coli* particles. Inset: the time-dependent particle number on the bottom when 100 particles are added to the solution. (**b**) The time-dependent *I_ds_* change when the graphene device was exposed to 100 CFU/mL *E. coli*. (**c**) The ∆*I_ds_* vs time when the simulation was performed with 11,950 *E. coli* particles. Inset: the time-dependent particle number on the bottom when 11,950 particles are added to the solution. (**d**) The time-dependent ∆*I_ds_* when the graphene device was exposed to 10^4^ CFU/mL *E. coli*. The dashed straight lines in red color represent the slopes at different rise periods in each figure.

As shown in Figure 9a, when the simulation was performed with 100 *E. coli* particles in the solution, both the number of bacteria on the graphene surface and ∆*I_ds_* exhibited time-dependent change. The number of bacteria attached onto the graphene surface increased slowly from 0 to 30 s, with faster increase from 40 to 120 s. Most *E. coli* particles (91) reached the graphene surface by 120 s. This behavior can be explained as follows. The bacterial motion in solution is affected by the gravitational force, Brownian force, drag force and swimming force in the first 30 s. When the *E. coli* particles are close to the graphene surface, the binding force between the sensing probe and bacteria could dominate the motion of *E. coli*, causing the number of bacteria on the graphene surface to increase fast. Accordingly, the ∆*I_ds_* increases gradually, which is consistent with the change of the number of bacteria on the graphene surface. The simulated ∆*I_ds_* is smaller than the experimental result as shown in Figure 9b, which may be due to the number of bacteria attached onto the graphene surface being larger in the experiment than that in the simulation. Moreover, a negative peak is seen in the first several seconds in Figure 9b, which is attributed to the disturbance from the addition of bacterial solution. This disturbance could cause the bacterial particles to reach the graphene surface in a shorter time; therefore, the obvious increase of the ∆*I_ds_* in the first several seconds in the experiment.

As shown in Figure 9c, for the simulation with a higher concentration of *E. coli* (11,950 *E. coli* particles in the solution), a slow increase in the number of bacterial particles on the graphene surface is seen in the first 8 s, followed by a much faster increase until 30 s, affected by the binding force. The rate is slower again from 30 to 90 s with saturation happening afterwards until 120 s. Accordingly, the calculated ∆*I_ds_* is consistent with the change in the number of bacteria on the graphene surface. Similarly, a negative peak is observed in the first several seconds, and the simulated ∆*I_ds_* is smaller than the experimental value, as shown in Figure 9d, which is attributed to the disturbance from the addition of bacterial solution and the larger number of bacteria reaching the graphene surface in the experiment. This simulation also suggests that the ∆*I_ds_* could reach saturation rapidly with the addition of high concentration of bacterial particles (larger than 11,950 particles) to the electrolyte.

We then compare the slope of the simulated and experimental current vs. time curve as shown in Table S1. The rise period is defined in Figure 9. The first 30 s of simulation was different from that of the experiment. The source-drain current in the simulation increased slightly during the first 30 s, which is ascribed to the slight increase of bacterial number on graphene surface. However, in the experiment, the addition of bacterial solution was performed by pipetting, which could cause a disturbance to the bacterial motion in the solution as the bacterial solution was squeezed from the pipette tip. Therefore, the slopes of the simulated and experimental current vs. time curve have some differences. The signal response is fast in the experiment work for low concentration (10^2^ CFU/mL), indicated by the slope value in the first rise period. The larger value from the experiment is attributed to more particles reaching the graphene surface in the experiment. At high concentration (11,950 CFU/mL), the slopes in different rise periods from the experiment and simulation are at the same level, except for a slow increase of the ∆*I_ds_* within a very short rise period. For the first rise period (0–30 s) and the second period (31–90 s), the slopes from the simulation and experiment are of the same order. The above analysis on the slope suggests that the simulation fits the experiment well. Additionally, a 2-stage response can be found in both low- and high-concentration cases.

**Figure 10:**
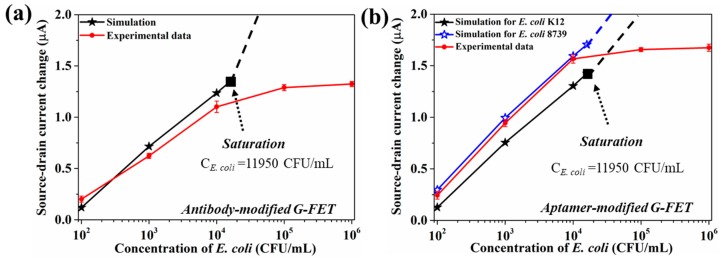
(**a**) The relationship between the source-drain current change and the concentration of *E. coli* from the experiment (5 devices) and the simulation for the aptamer-modified G-FET. (**b**) The relationship between the source-drain current change and the concentration of *E. coli* from the experiment and the simulation for the antibody-modified G-FET.

Next, we discuss the *E. coli* concentration-dependent ∆*I_ds_* and compare the simulation and experiment results. Figure 10 shows the ∆*I_ds_* of both the antibody-modified and aptamer-modified G-FETs for different concentrations of *E. coli* solution. According to the simulation results, the number of *E. coli* cells reaching the graphene surface is about 10,000 CFU/mL with the addition of 11,950 CFU/mL *E. coli*. When the graphene channel is completely occupied by *E. coli* and all these *E. coli* cells could induce holes in the graphene channel, only about 10,000 *E. coli* cells could attach to the surface of graphene, even when adding a higher concentration of *E. coli* solution. When higher concentration of *E. coli* (10^5^ and 10^6^ CFU/mL) is added, the number of *E. coli* reaching the graphene surface is still 10,000 CFU/mL, implying the attainment of saturation. As shown in Figure 10a, for the antibody-modified G-FET, the ∆*I_ds_* obtained from the experiment (red line, *n* = 5) is slightly smaller than that of the simulation result (black line). Some antibody molecules may lose their bio-activities, resulting in a smaller electrical response. The saturation of the source-drain current for the aptamer-modified G-FET happens with the addition of 11,950 CFU/mL *E. coli* (black line in Figure 10b). The electrical response from the experiment (red line, *n* = 5) is similar as in the simulation results. However, the value of ∆*I_ds_* is larger in the simulation because of the use of *E. coli* K12 as the target in the simulation, in contrast to *E. coli* 8739 as the target in the aptamer-related experiment. We assumed that *E. coli* 8739 might carry more surface charge than *E. coli* K12. To date, there is no report on the surface charge of *E. coli* 8739. Therefore, we calculated the value of the positive charge induced by single *E. coli* 8739 cell. When the simulation results were consistent with those of the experiment, the value might be about 2062. By using Equation (12), the surface charge of *E. coli* 8739 was evaluated as ~−3.59 × 10^−16^ C. Based on the experiment and the simulation results, the surface charge properties of the target could be evaluated.

**Figure 11:**
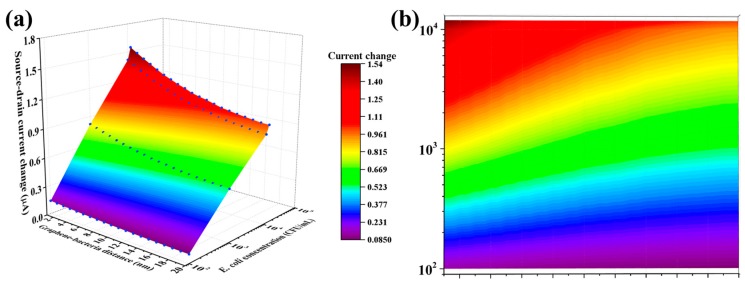
(**a**) 3D color map of the relationship between the source-drain current change, the graphene-bacteria distance and *E. coli* concentration. (**b**) Flat color map of the source-drain current change at different graphene-bacteria distance and *E. coli* concentration.

Although there are some gaps between the experiment results and the simulation, the simulation results still give us a deeper understanding of the operation of G-FET biosensors designed for high performance and provide a reference for evaluating the sensing performance. Figure 11 shows a color map of the value of the source-drain current change for different graphene-bacteria distance and *E. coli* concentration. Smaller graphene-bacteria distances (smaller height of the sensing probe) and higher bacterial concentrations give larger source-drain current change. Overall, this study provides a comprehensive understanding of the target motion in the electrolyte and surface charge induced by the target (*E. coli*). The established relationship between the source-drain current and graphene-bacterial distance here could help to design new graphene biosensors with excellent sensing performance. Since G-FETs are a material-specific subset of bio field effect transistors (BioFETs) with silicon and other materials serving as channels [23,24], the present modeling study would benefit BioFET design in general for various applications.

## 5. Conclusions

We have modeled and simulated the operation of G-FET biosensors, including the bacterial motion in the electrolyte and the surface charge induced by the charged bacterial particles. The charged *E. coli* particle and its motion at different times, functionalization with differently sized sensing probes and surface charge induced by adding *E. coli* cells at different concentrations were carefully investigated. The graphene-bacteria distance, defined by the size of the sensing probe, is found to play a key role in improving the sensing performance of the biosensors since it leads to more efficient induced surface charge and a resultant larger electrical response. More importantly, the relationship between the source-drain current, the graphene-bacteria distance, and the concentration of the bacterial particles was established. Smaller graphene-bacteria distances and higher bacterial concentrations yield larger changes of source-drain current of the biosensor. This relationship can help to predict the sensing performance of the G-FET biosensor. This study can serve as a guideline for the design and optimization of G-FET biosensors in the future.

## Figures and Tables

**Table 1:** The counted number of the particles at different concentrations on the bottom after 120 s.

Concentration CFU/mL (Added)	Concentration CFU/mL (Bottom)
10^2^	91 (91%)
10^3^	973 (97.3%)
10^4^	9154 (91.54%)
11,950	10,000 (91.32%)
10^5^	95,578 (95.58%)
10^6^	917,045 (91.7%)

## Supplementary Materials

The following are available online at http://www.mdpi.com/1424-8220/18/6/1715/s1. Figure S1: Motion of bacteria at different concentrations. Figure S2: Transfer curves of graphene device before and after exposure to bacterial solution. Table S1: The slope from the simulation and experiment during the rise period.

## References

[1] Novoselov K.S., Geim A.K., Morozov S.V., Jiang D., Zhang Y., Dubonos S.V., Grigorieva I.V., Firsov A.A. (2004). Electric field effect in atomically thin carbon films. Science.

[2] Geim A.K., Novoselov K.S. (2007). The rise of graphene. Nat. Mater..

[3] Fu W.Y., Jiang L., van Geest E.P., Lima L.M.C., Schneider G.F. (2017). Sensing at the surface of graphene field- effect transistors. Adv. Mater..

[4] Ohno Y., Maehashi K., Yamashiro Y., Matsumoto K. (2009). Electrolyte-gated graphene field-effect transistors for detecting pH and protein adsorption. Nano Lett..

[5] Ang P.K., Chen W., Wee A.T.S., Loh K.P. (2008). Solution-gated epitaxial graphene as pH sensor. J. Am. Chem. Soc..

[6] Wu G.F., Tang X., Meyyappan M., Lai K.W.C. (2017). Doping effects of surface functionalization on graphene with aromatic molecule and organic solvents. Appl. Surf. Sci..

[7] Yang G.H., Zhu C.Z., Du D., Zhu J.J., Lin Y.H. (2015). Graphene-like two-dimensional layered nanomaterials: Applications in biosensors and nanomedicine. Nanoscale.

[8] Georgakilas V., Tiwari J.N., Kemp K.C., Perman J.A., Bourlinos A.B., Kim K.S., Zboril R. (2016). Noncovalent functionalization of graphene and graphene oxide for energy materials, biosensing, catalytic, and biomedical applications. Chem. Rev..

[9] Akbari E., Buntat Z., Nilashi M., Afroozeh A., Farhange Y., Zeinalinezhad A. (2016). ISVR modeling of an interferon gamma (IFN-γ) biosensor based on graphene. Anal. Methods.

[10] Karimi H., Rahmani R., Mashayekhi R., Ranjbari L., Shirdel A.H., Haghighian N., Movahedi P., Hadiyan M., Ismail R. (2014). Analytical development and optimization of a graphene-solution interface capacitance model. Beilstein J. Nanotechnol..

[11] Karimi H., Yusof R., Rahmani R., Hosseinpour H., Ahmadi M.T. (2014). Development of solution-gated graphene transistor model for biosensors. Nano Res. Lett..

[12] Wu G.F., Tang X., Lin Z.H., Meyyappan M., Lai K.W.C. The effect of ionic strength on the sensing performance of liquid-gated biosensors. Proceedings of the IEEE 17th International Conference on Nanotechnology (IEEE-NANO).

[13] Wu G.F., Dai Z.W., Tang X., Lin Z.H., Lo P.K., Meyyappan M., Lai K.W.C. (2017). Graphene field-effect transistors for the sensitive and selective detection of *Escherichia coli* using pyrene-tagged DNA aptamer. Adv. Healthc. Mater..

[14] Chaumeil F., Crapper M. (2013). Using the DEM-CFD method to predict Brownian particle deposition in a constricted tube. Particuology.

[15] Kim M.M., Zydney A.L. (2004). Effect of electrostatic, hydrodynamic, and Brownian forces on particle trajectories and sieving in normal flow filtration. J. Colloid Interface Sci..

[16] Furlani E.P. (2006). Analysis of particle transport in a magnetophoretic microsystem. J. Appl. Phys..

[17] Chen Y., Li P., Huang P.H., Xie Y., Mai J.D., Wang L., Nguyen N.T., Huang T.J. (2014). Rare cell isolation and analysis in microfluidics. Lab Chip.

[18] Davis M.E., McCammon J.A. (1990). Electrostatics in biomolecular structure and dynamics. Chem. Rev..

[19] Fogolari F., Brigo A., Molinari H. (2002). The Poisson-Boltzmann equation for biomolecular electrostatics: A tool for structural biology. J. Mol. Recognit..

[20] Debye P., Huckel E. (1923). de la Theorie des Electrolytes. I. Abaissement du Point de Congelation et Phenomenes Associes. Physik. Z..

[21] Magnusson K.E., Davies J., Grundstrom T., Kihlstrom E., Normark S. (1980). Surface charge and hydrophobicity of *Salmonella*, *E. coli*, *Gonococci* in relation to their tendency to associate with animal cells. Scand. J. Infect. Dis. Suppl..

[22] Giubileo F., Bartolomeo A.D. (2017). The role of contact resistance in graphene field-effect devices. Prog. Surf. Sci..

[23] Rim T., Kim K.Y., Baek C.H., Jeong Y.H., Lee J.S., Meyyappan M. (2014). Silicon nanowire biologically sensitive field effect transistors: Electrical characteristics and applications. J. Nanosci. Nanotechnol..

[24] Moon D.I., Han J.W., Meyyappan M. (2016). Comparative study of field effect transistor biosensors. IEEE Trans. Nanotechnol..

