# Supplementary material for: Simulation of Graphene Field-Effect Transistor Biosensors for Bacterial Detection
Source: Sensors (Basel). 2018 May 25;18(6):1715. doi: 10.3390/s18061715 (PMC6021923; doi:10.3390/s18061715)
Supplement: Supplementary file 1 [file sensors-18-01715-s001.pdf]

Simulation of Graphene Field-Effect Transistor Biosensors for Bacterial Detection

Guangfu Wu [1,2,3], M. Meyyappan [4] and King Wai Chiu Lai [1,2,*]

1 Department of Mechanical and Biomedical Engineering, City University of Hong Kong, 83 Tat Chee Avenue, Kowloon, Hong Kong, China; guangfuwu1987@gmail.com

2 Centre for Robotics and Automation, City University of Hong Kong, 83 Tat Chee Avenue, Kowloon, Hong Kong, China; kinglai@cityu.edu.hk

3 Shanghai Key Laboratory of Modern Optical System, Engineering Research Center of Optical Instrument and System (Ministry of Education), University of Shanghai for Science and Technology, Shanghai

4 NASA Ames Research Center, Moffett Field, CA, USA; m.meyyappan@nasa.gov

Contents

1. Motion of bacteria at different concentrations

2. Transfer curves of graphene device before and after exposure to bacterial solution

3. The slope from the simulation and experiment during the rise period.

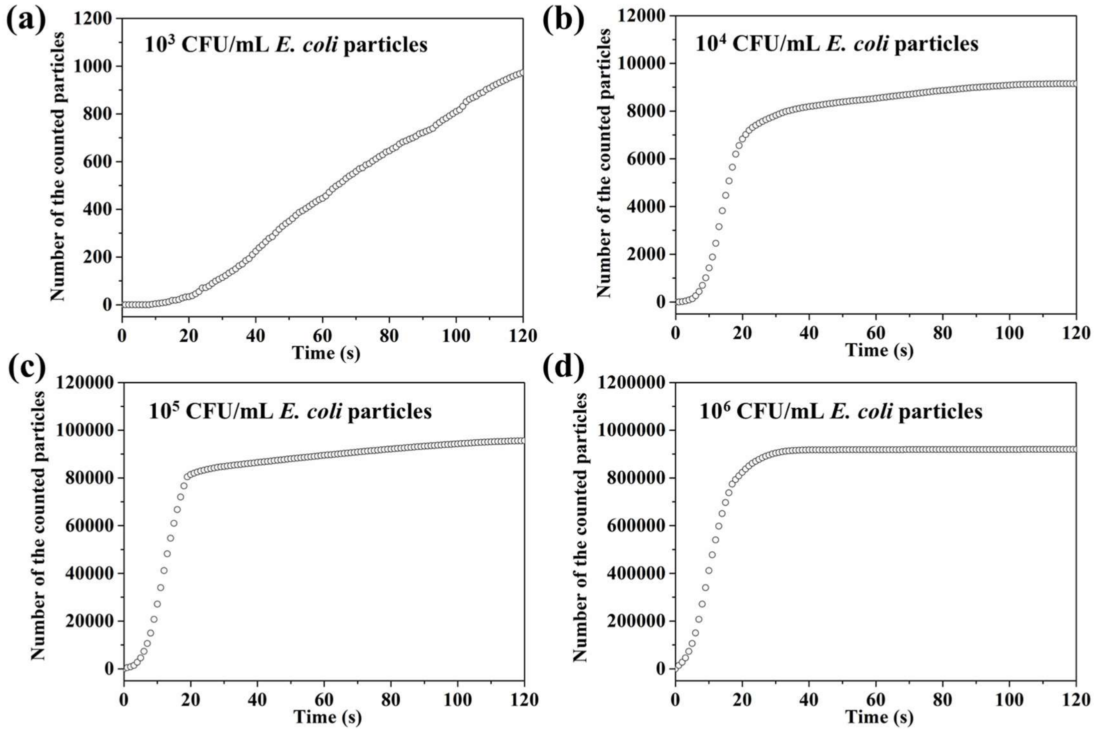

Figure S1. (a) The bacterial distribution from 0 to 120 s when 10^3 CFU/mL *E. coli* was simulated. (b) The bacterial distribution from 0 to 120 s when 10^4 CFU/mL *E. coli* was simulated. (c) The bacterial distribution from 0 to 120 s when 10^5 CFU/mL *E. coli* was simulated. (d) The bacterial distribution from 0 to 120 s when 10^6 CFU/mL *E. coli* was simulated.

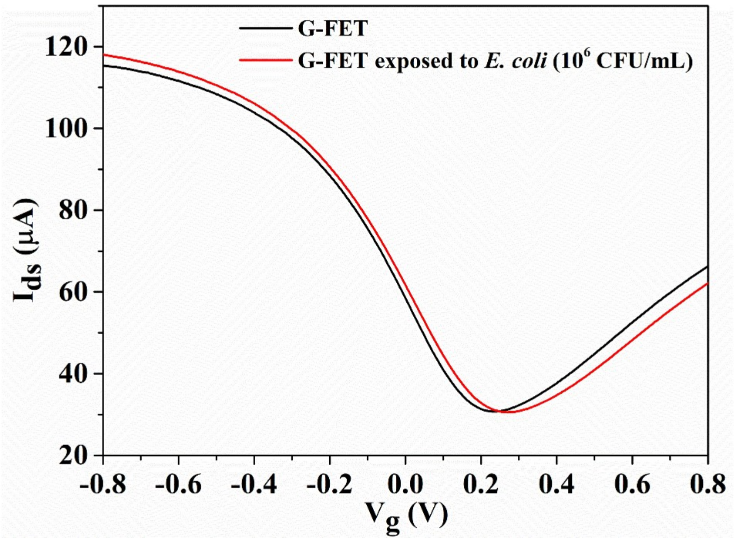

Figure S2. The transfer characteristics of the G-FET (black) and 10^6 CFU/mL of *E. coli* treated G-FET (red).

Table S1. The slope from the simulation and experiment during the rise period.

**Slope (μA/s) for the concentration of 100 CFU/mL**	**Simulation**	**Experiment**
For the first rise period (0 - 30s)	$3.17*10^{-4}$	$4.6*10^{-3}$
For the second rise period (31s - 120s)	$1.36*10^{-3}$	$7.33*10^{-4}$
**Slope (μA/s) for the concentration of 11950 CFU/mL**	**Simulation**	**Experiment**
For the first rise period (0 - 30s)	$8.67*10^{-2}$	$5.10*10^{-2}$
For the second rise period (31s - 90s)	$2.74*10^{-3}$	$6.24*10^{-3}$
For the saturation period (91s - 120s)	$7.3*10^{-4}$	$7.77*10^{-4}$
